# Multisensory integration, learning, and the predictive coding hypothesis

**DOI:** 10.3389/fpsyg.2014.00257

**Published:** 2014-03-24

**Authors:** Nicholas Altieri

**Affiliations:** ISU Multimodal Language Processing Lab, Department of Communication Sciences and Disorders, Idaho State UniversityPocatello, Idaho, USA

**Keywords:** predictive coding, Bayesian inference, audiovisual speech integration, EEG, parallel models

The multimodal nature of perception has generated several questions of importance pertaining to the encoding, learning, and retrieval of linguistic representations (e.g., Summerfield, 1987; Altieri et al., 2011; van Wassenhove, 2013). Historically, many theoretical accounts of speech perception have been driven by descriptions of auditory encoding; this makes sense because normal-hearing listeners rely predominantly on the auditory signal. However, from both evolutionary and empirical standpoints, comprehensive neurobiological accounts of speech perception must account for interactions across sensory modalities and the interplay of cross-modal and articulatory representations. These include auditory, visual, and somatosensory modalities.

In a recent review, van Wassenhove (2013) discussed key frameworks describing how visual cues interface with the auditory modality to improve auditory recognition (Sumby and Pollack, 1954), or otherwise contribute to an illusory percept for mismatched auditory-visual syllables (McGurk and MacDonald, 1976). These frameworks encompass multiple levels of analysis. Some of these higher cognitive processing models that discuss parallel processing (Altieri and Townsend, 2011) or the independent extraction of features from the auditory and visual modalities (Massaro, 1987, Fuzzy Logical Model of Perception), early feature encoding (van Wassenhove et al., 2005), and encoding/timing at the neural level (Poeppel et al., 2008; Schroeder et al., 2008).

This commentary on van Wassenhove (2013) will examine predictive coding hypotheses as one theory for how visemes are matched with auditory cues. Crucially, a hypothesized role shall be emphasized for cross-modal neural plasticity and multisensory learning in reinforcing the sharing of cues across modalities into adulthood.

## Predictive encoding and fixed priors

A critical question in speech research concerns how time-variable signals interface with internal representations to yield a stable percept. Although speech signals are highly variable (multiple talkers, dialects, etc.), our percepts appear stable due to dimensionality reduction. These questions become even more complex in multisensory speech perception since we are now dealing with the issue of how visual speech gestures coalesce with the auditory signal as the respective signals unfold at different rates and reach cortical areas at different times. In fact, these signals must co-occur within an optimal spatio-temporal window to have a significant probability of undergoing integration (Conrey and Pisoni, 2006; Stevenson et al., 2012).

The *predictive coding hypothesis* incorporates these aforementioned observations to describe integration in the following ways: (1) Temporally congruent auditory and visual inputs will be processed by cortical integration circuitry, (2), internal representations (“fixed Bayesian priors”) are compared and matched against the inputs, and (3) hypotheses about the intended utterance are actively generated. van Wassenhove et al.'s (2005) EEG study exemplified key components of the visual predicative coding hypothesis. When presented with auditory and visual syllables in normal conversational settings, the visual signal leads the auditory by tens or even hundreds of milliseconds. Thus, featural information in the visual signal constrains predictions about the content of the auditory signal. The authors showed that early visual speech information speeds-up auditory processing, as evidenced by temporal facilitation in the early auditory ERPs. This finding was interpreted as a reduction in the residual error in the auditory signal by the visual signal. One promising hypothesis is that visual information interacts with the auditory cortex in such a way that it modulates excitability in auditory regions via oscillatory phase resetting (Schroeder et al., 2008). Predictive coding hypotheses may also be extended to account for broad classes of stimuli including speech and non-speech, and matched and mismatched signals—all of which have been shown to evoke early ERPs associated with visual prediction (Stekelenburg and Vroomen, 2007).

## Fixed priors

Hypothetically, visual cues can provide predictive information so long as they precede the auditory stimulus and provide reliable cues (see Nahorna et al., 2012). A critical issue pertaining to visual predictive coding, then, relates to the “rigidity” of the internal rules (fixed priors). van Wassenhove (2013) discussed research suggesting the stability of priors/representations that are innate or otherwise become firmly established during critical developmental periods (Rosenblum et al., 1997; Lewkowicz, 2000). Lewkowicz (2000) argued that the ability to detect multisensory synchrony and match “duration and rate” are established early in life. In the domain of speech, Rosenblum and colleagues have argued that infants are sensitive to the McGurk effect and also to matched vs. mismatched articulatory movements and speech sounds.

While these studies suggest some rigidity of priors, I would emphasize that prior probabilities or “internal rules” remain malleable into adulthood. This adaptive perspective finds support among Bayesian theorists who argue that priors are continually updated in light of new evidence. Research indicates that differences in the ability to detect subtle auditory-visual asynchronies changes even into early adulthood (Hillock et al., 2011). Additionally, perceptual learning and adaptation techniques can alter priors in such a way that perceptions of asynchronies are modified via practice (Fujisaki et al., 2004; Vatakis et al., 2007; Powers et al., 2009) or experience with a second language (Navarra et al., 2010). Importantly, continual updating of “fixed” priors allows adult perceivers to (re)learn, fine tune, and adapt to multimodal signals across listening conditions, variable talkers, and attentional loads. van Wassenhove (2013) discussed how subjects can “automatically” match pitch and spatial frequency patterns (Evans and Treisman, 2010). This certainly shows that subjects can match auditory and visual information based on prior experience. Altieri et al. (2013) have also shown that adults can learn to match auditory and visual patterns more efficiently after only one day of practice! Reaction times and EEG signals indicated rapid learning and higher integration efficiency after only 1 h of training, followed by a period of gradual learning that remained stable over 1 week.

**Figure 1:**
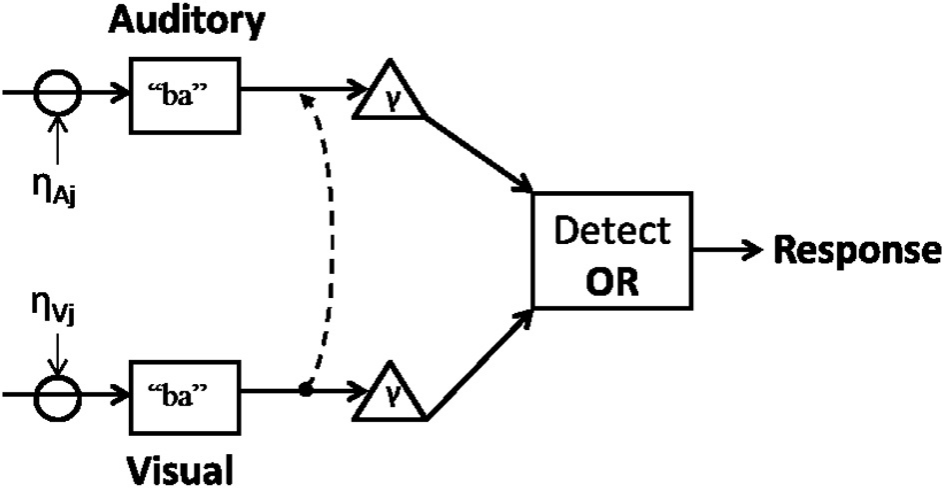
**Inputs interact with noise while evidence for a category (e.g., “ba”) accumulates toward threshold (γ)**. Once enough information in either modality reaches threshold, a decision is made (e.g., “ba” vs. “da”). Visual information interacts with auditory cortical regions (dotted line) leading to updated priors. This model does not rule out the possibility that auditory cues can reciprocally influence viseme recognition.

Such findings appear consistent with a unified parallel framework where visual information influences auditory processing and where visual predictability can be reweighted through learning. Figure 1 represents an attempt to couch predictive coding within adaptive parallel accounts of integration.

## References

[B1] Altieri N. Pisoni D. B. Townsend J. T. (2011). Behavioral, clinical, and neurobiological constraints on theories of audiovisual speech integration: a review and suggestions for new directions. Seeing Perceiving 24, 513–539 10.1163/187847611X595864 21968081 PMC3293210

[B2] Altieri N. Stevenson R. A. Wallace M. T. Wenger M. J. (2013). Learning to associate auditory and visual stimuli: capacity and neural measures of efficiency. Brain Topogr. [Epub ahead of print]. 10.1007/s10548-013-0333-7 24276220 PMC4043950

[B3] Altieri N. Townsend J. T. (2011). An assessment of behavioral dynamic information processing measures in audiovisual speech perception. Front. Psychol. 2:238 10.3389/fpsyg.2011.00238 21980314 PMC3180170

[B4] Conrey B. Pisoni D. B. (2006). Auditory-visual speech perception and synchrony detection for speech and nonspeech signals. J. Acoust. Soc. Am. 119, 4065 10.1121/1.2195091 16838548 PMC3314884

[B5] Evans K. K. Treisman A. (2010). Natural cross-modal mappings between visual and auditory features. J. Vis. 10:6 10.1167/10.1.6 20143899 PMC2920420

[B6] Fujisaki W. Shimojo S. Kashino M. Nishida S. (2004). Recalibration of audiovisual simultaneity. Nat. Neurosci. 7, 773–778 10.1038/nn1268 15195098

[B7] Hillock A. R. Powers A. R. Wallace M. T. (2011). Binding of sights and sounds: age-related changes in audiovisual temporal processing. Neuropsychologia 49, 461–467 10.1016/j.neuropsychologia.2010.11.041 21134385 PMC3140703

[B8] Lewkowicz D. J. (2000). The development of inter-sensory temporal perception: an epignetic systems/limitations view. Psychol. Bull. 162, 281–308 10.1037/0033-2909.126.2.281 10748644

[B22] Massaro D. W. (1987). Speech perception by ear and eye, in Hearing by Eye: The Psychology of Lip-Reading, eds Dodd B. Campbell R. (Hillsdale, NJ: Lawrence Erlbaum), 53–83

[B9] McGurk H. MacDonald J. W. (1976). Hearing lips and seeing voices. Nature 264, 746–748 10.1038/264746a0 1012311

[B23] Nahorna O. Berthommier F. Schwartz J. L. (2012). Binding and unbinding the auditory and visual streams in the McGurk effect. J. Acoust. Soc. Am. 132, 1061–1077 10.1121/1.4728187 22894226

[B10] Navarra J. Alsius A. Velasco I. Soto-Faraco S. Spence C. (2010). Perception of audiovisual speech synchrony for native and non-native language. Brain Res. 1323, 84–93 10.1016/j.brainres.2010.01.059 20117103

[B11] Poeppel D. Idsardi W. J. van Wassenhove V. (2008). Speech perception at the interface of neurobiology and linguistics. Philos. Trans. R. Soc. Lond. B Biol. Sci. 363, 1071–1086 10.1098/rstb.2007.2160 17890189 PMC2606797

[B12] Powers A. R. 3rd. Hillock A. R. Wallace M. T. (2009). Perceptual training narrows the temporal window of multisensory binding. J. Neurosci. 29, 12265–12274 10.1523/JNEUROSCI.3501-09.2009 19793985 PMC2771316

[B13] Rosenblum L. Schmuckler M. A. Johnson J. A. (1997). The McGurk effect in infants. Percept. Psychophys. 59, 347–357 10.3758/BF03211902 9136265

[B14] Schroeder C. Lakatos P. Kajikawa Y. Partan S. Puce A. (2008). Neuronal oscillations and visual amplification of speech. Trends Cogn. Sci. 12, 106–113 10.1016/j.tics.2008.01.002 18280772 PMC3987824

[B15] Stekelenburg J. J. Vroomen J. (2007). Neural correlates of multisensory integration of ecologically valid audiovisual events. J. Cogn. Neurosci. 19, 1964–1973 10.1162/jocn.2007.19.12.1964 17892381

[B16] Stevenson R. A. Zemtsov R. K. Wallace M. T. (2012). Individual differences in the multisensory temporal binding window predict susceptibility to audiovisual illusions. J. Exp. Psychol. Hum. Percept. Perform. 38, 1517–1529 10.1037/a0027339 22390292 PMC3795069

[B17] Sumby W. H. Pollack I. (1954). Visual contribution to speech intelligibility in noise. J. Acoust. Soc. Am. 26, 212–215 10.1121/1.1907309

[B18] Summerfield Q. (1987). Some preliminaries to a comprehensive account of audio-visual speech perception, in The Psychology of Lip-Reading, eds Dodd B. Campbell R. (Hillsdale, NJ: LEA), 3–50

[B19] van Wassenhove V. (2013). Speech through ears and eyes: interfacing the senses with the supramodal brain. Front. Psychol. 4:388 10.3389/fpsyg.2013.00388 23874309 PMC3709159

[B20] van Wassenhove V. Grant K. W. Poeppel D. (2005). Visual speech speeds up the neural processing of auditory speech. Proc. Natl. Acad. Sci. U.S.A. 102, 1181–1186 10.1073/pnas.0408949102 15647358 PMC545853

[B21] Vatakis A. Navarra J. Soto-Faraco S. Spence C. (2007). Temporal recalibration during asynchronous audiovisual speech perception. Exp. Brain Res. 181, 173–181 10.1007/s00221-007-0918-z 17431598

